# The utility of flow sorting to identify chromosomes carrying a single copy transgene in wheat

**DOI:** 10.1186/s13007-016-0124-8

**Published:** 2016-04-25

**Authors:** Petr Cápal, Takashi R. Endo, Jan Vrána, Marie Kubaláková, Miroslava Karafiátová, Eva Komínková, Isabel Mora-Ramírez, Winfriede Weschke, Jaroslav Doležel

**Affiliations:** Institute of Experimental Botany, Centre of the Region Haná for Biotechnological and Agricultural Research, Šlechtitelů 31, 78371 Olomouc, Czech Republic; Faculty of Agriculture, Ryukoku University, 1-5 Yokotani, Seta Oe-cho, Otsu, Shiga 520-2194 Japan; Leibniz Institute of Plant Genetics and Crop Plant Research (IPK) Gatersleben, Corrensstrasse 3, 06466 Stadt Seeland, Germany

**Keywords:** Transgene localization, Flow cytometric sorting, Single chromosome amplification, *Triticum aestivum*, *Hordeum vulgare*, HvSUT1

## Abstract

**Background:**

Identification of transgene insertion sites in plant genomes has practical implications for crop breeding and is a stepping stone to analyze transgene function. However, single copy sequences are not always easy to localize in large plant genomes by standard approaches.

**Results:**

We employed flow cytometric chromosome sorting to determine chromosomal location of barley sucrose transporter construct in three transgenic lines of common wheat. Flow-sorted chromosomes were used as template for PCR and fluorescence in situ hybridization to identify chromosomes with transgenes. The chromosomes carrying the transgenes were then confirmed by PCR using DNA amplified from single flow-sorted chromosomes as template.

**Conclusions:**

Insertion sites of the transgene were unambiguously localized to chromosomes 4A, 7A and 5D in three wheat transgenic lines. The procedure presented in this study is applicable for localization of any single-copy sequence not only in wheat, but in any plant species where suspension of intact mitotic chromosomes suitable for flow cytometric sorting can be prepared.

**Electronic supplementary material:**

The online version of this article (doi:10.1186/s13007-016-0124-8) contains supplementary material, which is available to authorized users.

## Background

During the past 30 years, many cultivars of agricultural crops beneficial to humankind have been developed by means of genetic engineering, including plants resistant to herbicides, pests or viruses, bearing fruits with prolonged shelf life and products more suited for industrial processing [for review see 1]. Wheat ranks 5th in the commodities produced worldwide and is the second most-produced food crop occupying more than 50 % of the world crop area (http://faostat3.fao.org/). In the light of climate change and world population growth, future challenges for the increase of crop production have constantly been discussed. However, FAO statistics show that the wheat production is reaching a plateau and is severely affected by climate change. This is a consequence of a slowdown in wheat yield increase, accounting for only 0.5 % per year in the last decade [2].

Breeding improved cultivars with increased tolerance to adverse climatic conditions and with increased yield and quality could be facilitated by genetic engineering and introduction of beneficial genes from other organisms. The insertion site of a transgene is of great importance for the transgene function [3, 4] which is also influenced by its position on the chromosome, including the flanking DNA sequences [5]. However, transgene localization is not easy by routine approaches, like fluorescence in situ hybridization (FISH), or Southern blotting. A prevalent method for detection of transgenes in animals and plants is FISH, which has its pros and cons [6]. In barley and common wheat, FISH enables cytological localization of cDNAs, as short as 1.5 kb, on a chromosome or chromosomes that had already been known to carry the cDNAs [7, 8]. Although some authors succeeded in localizing transgenes on plant chromosomes using FISH [9–11], this approach has not become a routine application.

Weichert et al. [12] obtained transgenic lines (HOSUT) of hexaploid wheat carrying barley (*Hordeum vulgare*) sucrose transporter HvSUT1 (SUT) gene that is overexpressed under the control of the endosperm-specific Hordein B1 promoter (HO). The HOSUT lines were found to increase grain yield significantly as compared to control non-transformed plants [13]. However, the genomic location of the transgene in these lines was not known. In the present work we employed a novel approach for unambiguous identification of chromosomes carrying the transgene in three HOSUT lines. The protocol takes the advantage of the availability of a procedure for flow cytometric chromosome sorting in wheat and the fact that flow-sorted chromosomes are suitable as templates for PCR and FISH [14]. Moreover, a protocol has been developed recently for representative DNA amplification from single copies of chromosomes [15]. By combining these approaches we could assign the transgene to particular chromosomes in three HOSUT lines of wheat.

## Results and discussion

**Fig. 1:**
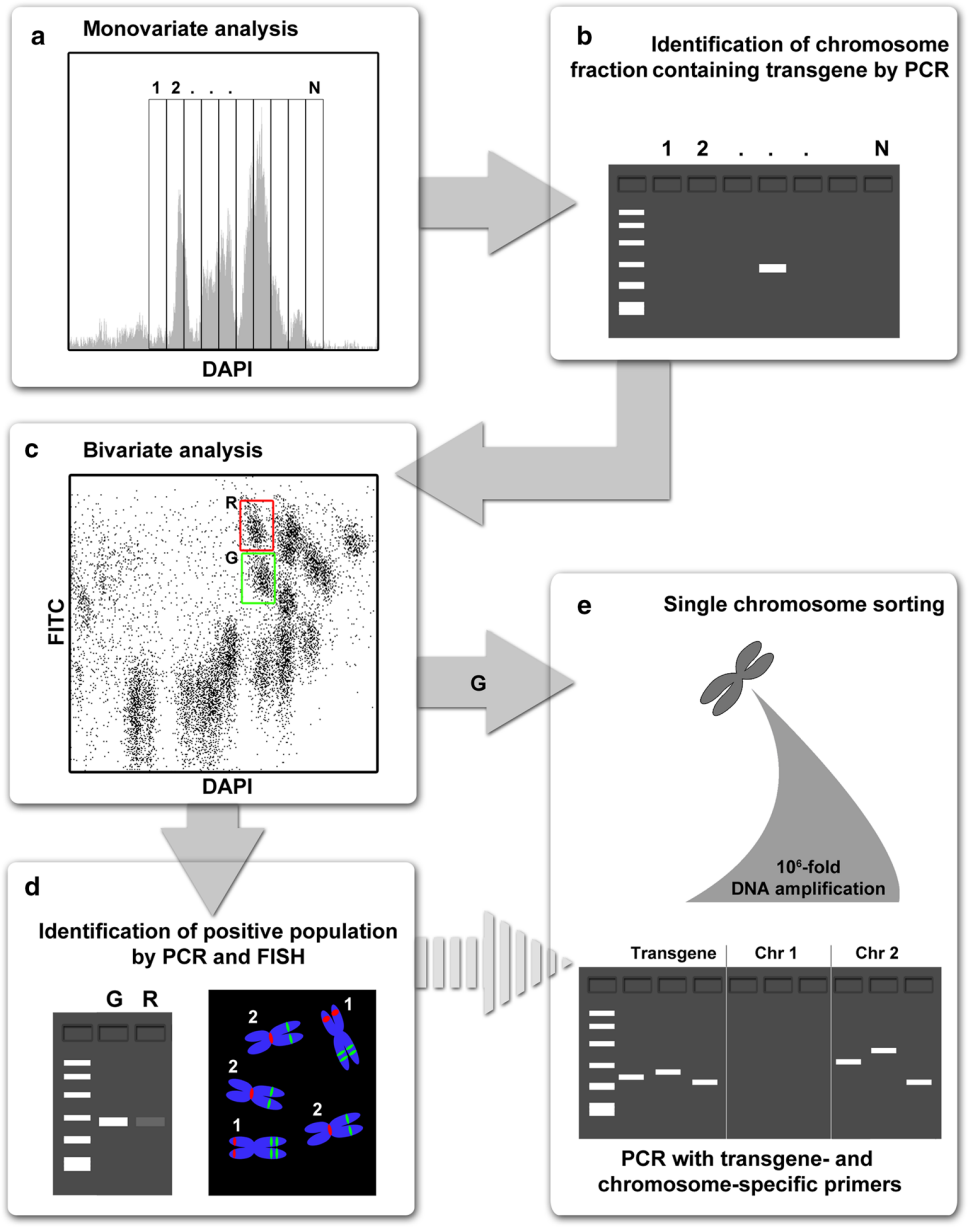
Experimental workflow. **a** Monovariate flow karyotype is dissected into small regions. From each region, 200 chromosomes are sorted. **b** Flow-sorted chromosomes are used as template for PCR with transgene-specific marker. **c** The region representing chromosome with the transgene identified on monovariate flow karyotype is dissected by sorting chromosomes from sort regions on bivariate flow karyotype. **d** From the sort gates on the bivariate flow karyotype, 100 chromosomes are sorted for PCR with transgene-specific marker. Moreover, 1000 chromosomes are sorted immediately afterwards onto the microscopic slides to identify flow-sorted chromosomes by FISH. **e** From the sort gate most enriched for the transgene-bearing chromosome (region *G* in this example), single chromosomes are sorted individually into PCR tubes. DNA of single-flow sorted chromosomes is amplified and resulting DNA is used as template for PCR to identify the presence of multiple transgene- and chromosome-specific sequences. This step unambiguously confirms the chromosome with integrated transgene

**Fig. 2:**
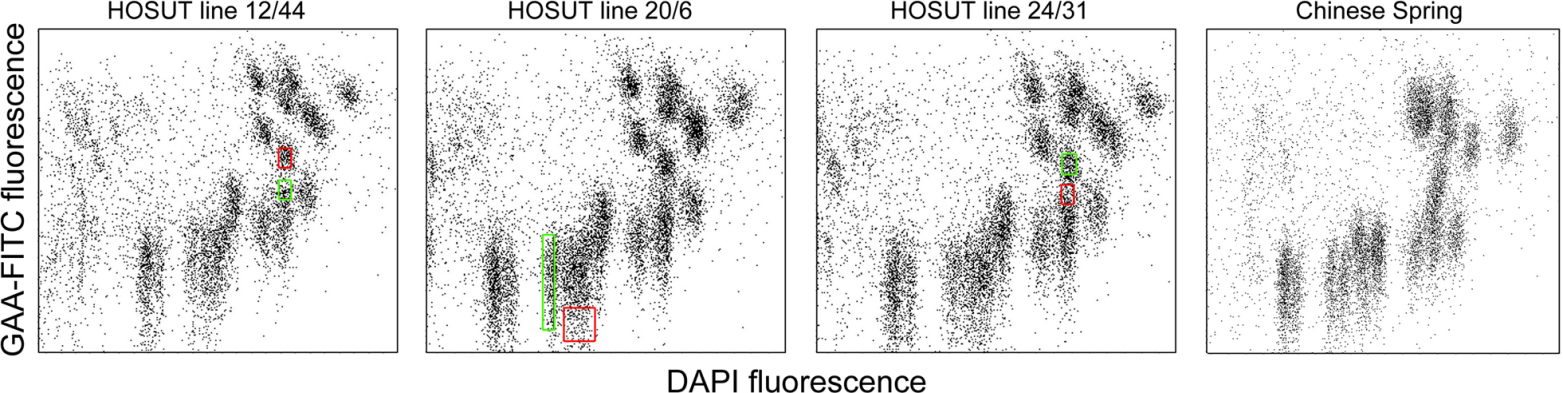
Bivariate flow karyotypes of three transgenic HOSUT lines of wheat obtained after the analysis of chromosomes with FITC-labelled (GAA)_n_ microsatellites and stained by DAPI. The position of *red* and *green* regions used to sort particular chromosomes is indicated. The *green* sort gate was found to represent chromosomes carrying transgene. Chromosomes were flow-sorted also from the neighboring population delineated by *red* gate and were used as a control. Although the transgene-bearing chromosome should not be included in this region, the sorted population could potentially be contaminated with transgene-bearing chromosomes due to similarity in chromosome size and DNA content

The experimental workflow is shown on Fig. 1. As the first step, we prepared liquid suspensions of intact mitotic chromosomes from all five lines of wheat (see “Methods” section) and analyzed them by flow cytometry. Monovariate flow karyotypes (histograms of relative fluorescence intensity) were obtained after the analysis of DAPI-stained chromosomes, and bivariate flow karyotypes obtained after the analysis of DAPI-stained chromosomes with FITC-labelled GAA microsatellites. We observed differences between flow karyotypes of the HOSUT lines and the model hexaploid wheat cultivar Chinese Spring. The alterations concerned the profiles of major composite peaks on monovariate flow karyotypes (Additional file 1: Figure S1) and the distribution of chromosome populations on bivariate flow karyotypes (Fig. 2). This observation reflected the differences in karyotypes (chromosome polymorphism) between the cultivar Certo, used to produce the HOSUT lines (data not shown), and Chinese Spring. On the other hand, flow karyotypes of the three HOSUT lines were indistinguishable from each other.

**Fig. 3:**
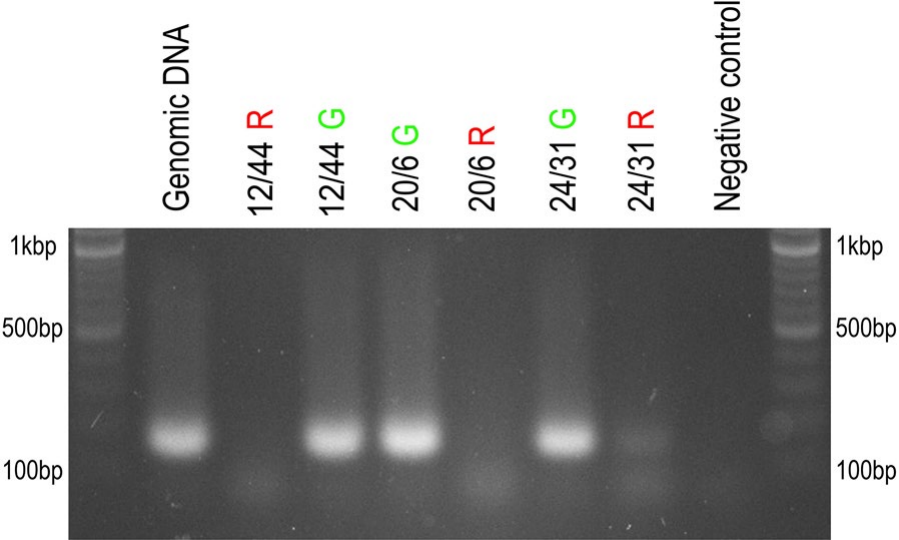
Agarose gel electrophoresis of PCR products obtained with primers for the transgene and DNA of chromosomes flow-sorted from three HOSUT lines using the *green* and *red* sort regions as shown in Fig. 1. The amplicon of HvSUT-RT (169 bp) was obtained with chromosomes sorted from the *green* sort region in all three HOSUT lines. When chromosomes were sorted from the *red* sort regions, no PCR amplification occurred for HOSUT 12/44 and HOSUT 20/6. However, a weak band was observed for HOSUT 24/31. Genomic DNA of the transgenic lines served as positive control

**Fig. 4:**
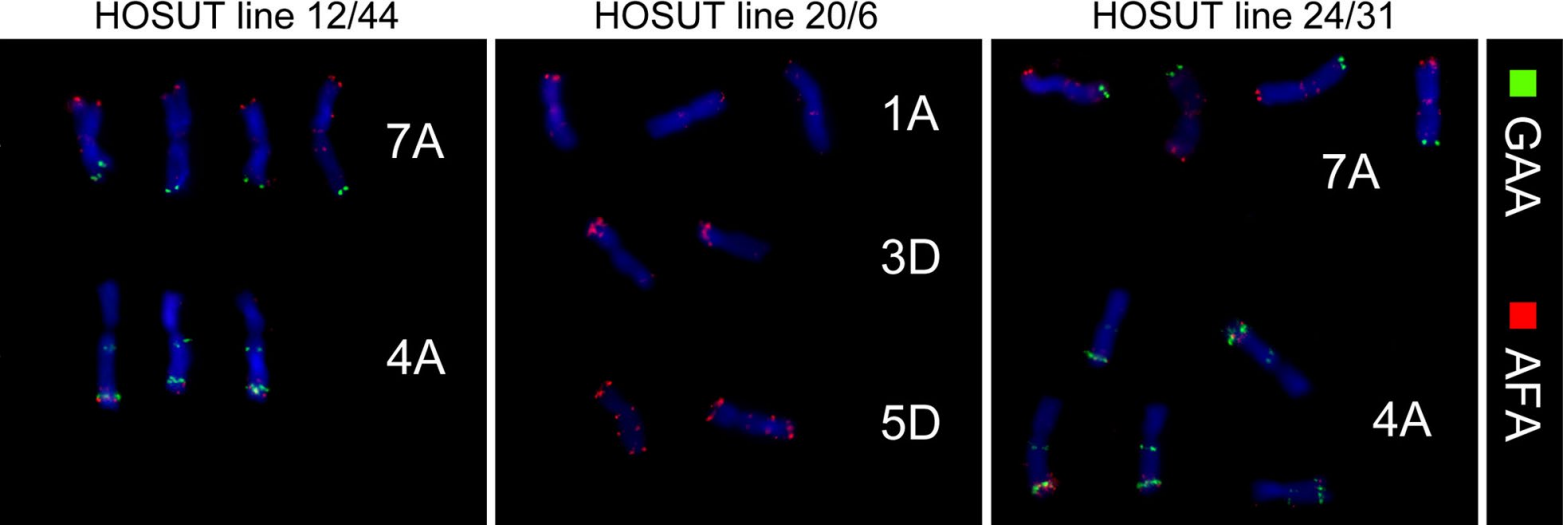
Representative images of chromosomes flow-sorted from three HOSUT lines using the *green* and *red* sort regions on bivariate flow karyotypes as shown in Fig. 2. FISH was done using probes for Afa-family (*red* signals) and GAA microsatellites (*green* signals). Chromosomes were counterstained with DAPI (*blue*)

In order to identify chromosomes carrying the transgenes, we first used the approach described by Vrána et al. [16]. Fractions of 200 chromosomes were sorted from different regions of monovariate flow karyotypes as shown on Additional file 1: Figure S1, and DNA of the sorted chromosomes was used as template for PCR. This analysis identified one region (sort gate) in each line as representing chromosomes bearing a transgene. As each region (sort gate) on a monovariate flow karyotype may represent more than one chromosome type, in the next step we sorted chromosomes from regions delineated on bivariate flow karyotypes (Fig. 2). The sort gates were designed to include chromosome populations corresponding to the positive sort gates on monovariate flow karyotypes. From these regions, and also from nearby regions, chromosomes were sorted into PCR tubes (100 chromosomes per tube) and immediately afterwards also onto microscopic slides (ca. 1000 chromosomes per slide). The results obtained by PCR with primers amplifying HvSUT-RT sequence (Fig. 3) and identification of chromosomes from sort gates for each transgenic line by FISH with probes targeting Afa-repeat family and GAA-microsatellites (Fig. 4) are summarized in Table 1.

**Table 1:** PCR and FISH analysis of chromosomes sorted from each of the sort gates in three HOSUT lines

Transgenic line	Sort gate^a^	PCR result	Chromosomes identified FISH^c^
HOSUT 12/44	Red	Negative	4A (92.65 %)
	Green	Positive	7A (90.90 %)
			4A (4.45 %)
HOSUT 20/6	Red	Negative	1A (63.75 %)
			3D (36.25 %)
	Green	Positive	5D (94.66 %)
			1A (5.33 %)
HOSUT 24/31	Red	Semi-positive^b^	7A (83.19 %)
			4A (12.39 %)
			2A (4.42 %)
	Green	Positive	4A (97.30 %)

^a^Sort gates delineated with green and red rectangles in Fig. 2

^b^A faint band was visible after agarose gel electrophoresis of PCR product

^c^More than 1000 chromosomes were examined in each sorted fraction in each line

FISH analysis showed that more than 90 % of chromosomes flow-sorted from the region defined by the green rectangle consisted of one type of chromosome in each of the HOSUT lines. This fact together with the results of PCR suggested that the transgene was located on chromosome 7A in HOSUT 12/44, on chromosome 5D in HOSUT 20/6 and on chromosome 4A in HOSUT 24/31. In the former two lines, the critical type of chromosome was not found among the chromosomes flow-sorted from the region defined by red rectangles. However, chromosome 4A was found to represent 12.39 % of chromosomes flow-sorted from the red region in HOSUT 24/31. This was probably due to the similarities in size and the amount of GAA-FITC fluorescence of chromosomes 4A and 7A. Due to this similarity, mixture of the two chromosomes 4A and 7A was also observed in the chromosome fraction sorted from the green region in HOSUT 12/44.

To confirm chromosomal locations of the transgene and avoid ambiguous results due to possible contamination of flow-sorted fraction by other chromosomes, PCR was done on DNA amplified from single flow-sorted chromosomes. As each time only one copy of chromosome is sorted, the DNA cannot be contaminated by other chromosomes. Five single chromosomes were sorted from the green sort regions of the HOSUT lines and their DNA was separately amplified using multiple displacement amplification (MDA). Out of the five sorted chromosomes, whole genome amplification was successful with three chromosomes in HOSUT 12/44, two chromosomes in HOSUT 20/6 and four chromosomes in HOSUT 24/31. The successful amplification was defined by the production of measurable amount of DNA after MDA and by the presence of at least one marker for the transgene and one marker for the wheat chromosome. The reason for occasional failure to amplify DNA from single chromosomes, which was observed previously [15] is not clear. One explanation is that a droplet with sorted chromosome lands on side wall of PCR tube and the chromosome is excluded from the MDA reaction. The amount of chromosomal DNA in successfully amplified samples ranged from 0.3 to 1.7 µg DNA.

**Fig. 5:**
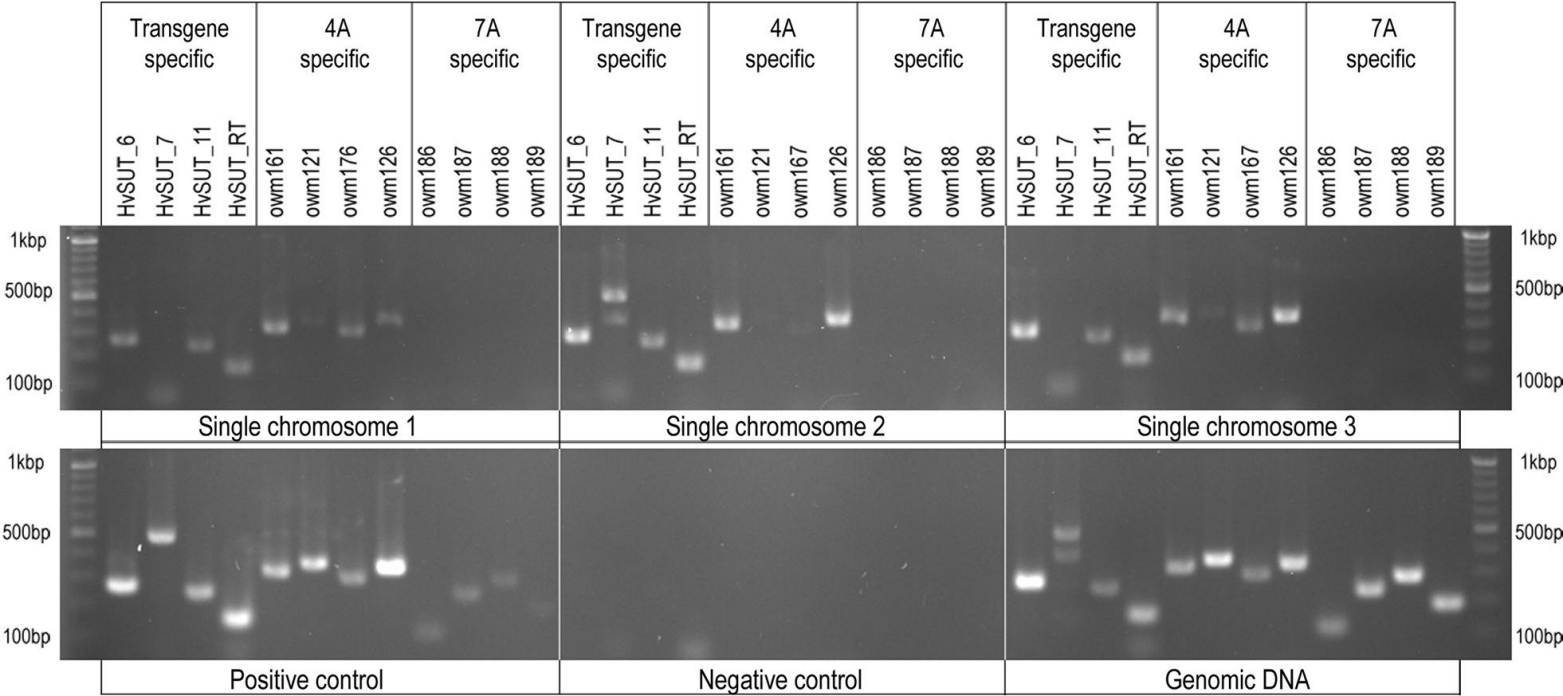
Agarose gel electrophoresis of PCR products obtained using DNA produced by multiple displacement amplification of three single chromosomes flow-sorted from the sort region representing chromosome 4A in HOSUT 24/31 line. PCR with primers for the four transgenes resulted in products of expected length. The same was true for the chromosome 4A-specific STS markers. Note that none of the chromosome 7A-specific markers was detected in the samples of single chromosome DNA. PCR with genomic DNA of HOSUT 24/31 as template detected both 4A and 7A chromosome-specific markers. PCR with the positive control (represented by 1000 chromosomes sorted from *green* sorting region and amplified) showed slight PCR bands of chromosome 7A, which reflects a minor contamination of the sorted chromosome 4A by chromosome 7A

Chromosome specificity of sequence tagged site (STS) markers used in this work to identify individual chromosomes was first tested using the euploid and corresponding nulli-tetrasomic lines of Chinese Spring (Additional file 2: Figure S2). The results confirmed that the markers were suitable for unambiguous identification of wheat chromosomes 1A, 4A, 5D and 7A. PCR analysis using both transgene- and chromosome-specific markers clearly confirmed chromosome location of transgenes as determined in the first part of this study. In case of HOSUT 24/31, where the location of the transgene was ambiguous, all four transgene markers were detected in DNA amplified from single chromosomes sorted from the green region (Fig. 5), and all four 4A-specific markers were also amplified in the same amplicons. None of the four 7A-specific markers was found in the same amplicons.

## Conclusions

Coupling PCR and FISH mapping using flow-sorted mitotic chromosomes as templates narrowed down the list of candidate chromosomes harboring the transgene to one or two chromosomes. PCR on DNA amplified from single flow-sorted chromosomes then unambiguously identified the chromosomes with the integrated transgene. If chromosome-specific PCR-based markers are available, mapping on single copy chromosomes could be an ultimate approach to assign single copy DNA sequences, including transgenes, to particular chromosomes. Moreover, the sequence assembly of amplicons from the chromosome could allow detecting the position of transgene insertion, if enough sequence information on the chromosome is available. However the main purpose of this work was to assign a transgene to particular chromosomes. The approach presented here is currently applicable to more than 25 plant species, which include important cereals and legumes [14] where liquid suspensions of mitotic chromosomes suitable for flow cytometric sorting can be prepared.

## Methods

### Plant material

We used German winter wheat cultivar Certo (*Triticum aestivum* L., 2n = 6x = 42, genome formula AABBDD) and its three transgenic lines, HOSUT 12/44, HOSUT 20/6 and HOSUT 24/31. The transgenic lines contain a single copy of the HvSUT1-cDNA (1894 bp) fused to the barley HorB1 promoter (550 bp) and the barley HorB1 terminator (1663 bp) [12]. We also used euploid and nullisomic–tetrasomic (Nt1A1B, Nt7A7B, Nt5D5B) lines of hexaploid wheat cultivar Chinese Spring (obtained from NBRP-wheat) to confirm the specificity of PCR markers to particular wheat chromosomes.

### Flow cytometric chromosome sorting

Cell cycle synchronization and metaphase accumulation of root tip meristem cells was performed as described previously [17], except for the formaldehyde fixation, which was shortened to 15 min. Isolated chromosomes were labelled by FISHIS (fluorescence in situ hybridization in suspension) using FITC-labeled GAA probe following the protocol of Giorgi et al. [18]. Flow cytometric analysis and sorting was done on BD FACSAria II high speed flow sorter equipped with 390 nm laser for DAPI excitation and 488 nm laser for FITC excitation. Sort gates were initially drawn on monovariate flow karyotypes of DAPI fluorescence (not shown) and subsequently on bivariate flow karyotypes of DAPI fluorescence versus GAA-FITC fluorescence as shown in Fig. 1.

### Fluorescence in situ hybridization (FISH)

For microscopic observations, 1000 chromosomes were sorted onto microscope slides from each of the sort regions. The slides were left to air-dry in the dark overnight. Then the preparations were used for FISH following the protocol of Kubaláková et al. [19] using a Cy5-labeled probe targeting Afa-family repeats, the chromosomes were already labeled by a GAA microsatellite probe during the FISHIS procedure.

### PCR

PCR was done using primers specific for the HOSUT transgene and for markers specific for candidate wheat chromosomes (Table 2). Of the four HOSUT primers, three were designed in the HvSUT1 region (accession no. AJ272309) and one in the HorB1 terminator region (accession no. FN643080). Wheat chromosome-specific markers were designed by Primer3 based on the chromosome sequences from the International Wheat Genome Sequencing Consortium (IWGSC), while preventing the primers from amplifying the sequence from the homoeologous chromosomes. PCR conditions were set as follows: initial denaturation 95 °C for 3 min, 35 cycles of 30 s denaturation at 95 °C, annealing at 58–62 °C (see Table 2 for T_a_ of the primer pairs) for 30 s and extension at 72 °C for 30 s, followed by final extension at 72 °C for 5 min. The amount of template DNA was 5 ng for each reaction. PCR products were run on 1.5 % agarose gels. PCR using 100 sorted chromosomes as template was conducted after a few freeze–thaw cycles to disintegrate the chromosomes and the initial denaturation step was prolonged for 7 min.

**Table 2:** List of PCR primers for the HOSUT transgene construct and PCR primers for wheat STS markers on chromosomes 1A, 4A, 7A and 5D

Name	Target	Forward primer sequence	Reverse primer sequence	Amplicon size (bp)	Annealing temperature (°C)
HvSUT_6	HOSUT1	AGCGGCGGCGGTCACTGACTG	CCAAAGGACGACACCCCAGCC	265	62
HvSUT_7	HorB1 terminator	ATTAATTCCTCCCCGACCCTGC	CAATGGAGACGGCGCGTGCAA	471	62
HvSUT_11	HOSUT1	GGCGGAACCCGCCGTGCAG	CCTGCGTCTTCCCCATCTGGAAGTA	241	62
HvSUT_RT	HOSUT1	CGGGCGGTCGCAGCTCGCGTCTATT	CATACAGTGACTCTGACCGGCACACA	169	62
Owm121	Chromosome 4A	ATTGCCGTCGCGAACTAGA	CGGGACGAGCTTGACGAT	351	60
Owm126	Chromosome 4A	CCAGTCAGAAATTATTATGAACCTATC	CGCTGTCTCGAGATTGGAGT	342	60
Owm161	Chromosome 4A	TTTTCAAGCAGGTTTTGTGC	TCACTTCTCTTCTTTGCGTTCA	324	60
Owm167	Chromosome 4A	TTTTCTTGGTCAGTATAACCTGTTTTT	TGAGCAGAGAAAAATTTCCAAG	285	60
Owm174	Chromosome 1A	GCATCCTAGTTTCTCTCTCAAGT	AACAAGATCACGAGCGAATTG	157	58
Owm175	Chromosome 1A	AAACCCCTGATACTCATGCG	GTTTCTTGTCATTCATGTCACTTGT	530	58
Owm176	Chromosome 1A	TTCCTGTCTGACTCCGCG	AACCACAACCGTCAACCG	104	58
Owm177	Chromosome 1A	GTAGTCTGCTCCCGAGGAAT	GTCTCTAACCATACATCCATGAAGT	192	58
Owm178	Chromosome 1A	CAACTTCTTCACATCCCGGAA	ATTTGGCCCTATGAGATATAATTACG	306	58
Owm179	Chromosome 1A	ACACTGTGATACCTCTAGATGTATG	CACATTGCCTATAAATTCTAAAAGGTC	425	58
Owm180	Chromosome 5D	CGGACGAGCAGCAGTACC	GCAGATCGGCATAAATTGAATGT	292	58
Owm181	Chromosome 5D	GGAGGTGTTCTAGGTGTACTTACT	AGAGCAATGTCAGAAGTCATCG	240	58
Owm182	Chromosome 5D	TCTCCACCTGCAGAGTCG	CATCAGGCCACAGTGTCAAT	119	58
Owm183	Chromosome 5D	TGTCCACACATTTCCCGTATG	AGTGGTGGATGTGGTTGCT	196	58
Owm184	Chromosome 5D	AGCATGCTCCCAAAGACTATTAC	GTTATGATGGTGGTAGCAATTTGA	400	58
Owm185	Chromosome 5D	GTGAACCTATATGACATCTTACCGG	GGGGCAGTTGTCAAGTATTGC	421	58
Owm186	Chromosome 7A	CTCTCTGTGGCCAATAGTGC	TCTATACCTCAACCCTACATCCA	112	58
Owm187	Chromosome 7A	GGCCACGAATTCCACAAGTA	CTATCGATCAACCAACCATCCA	229	58
Owm188	Chromosome 7A	GTACGAGTGCAGACAGTGTG	ACAATTAATTATACGCCCAGTTAAGC	282	58
Owm189	Chromosome 7A	CGTGCTTTCTTCTTCCTCCG	GCAGGTTAGTTTCTTGTGGTTG	185	58
Owm190	Chromosome 7A	CGCATGGACATTGTTCTAGTCA	GCACTTAGGCACGCTTGAG	517	58
Owm191	Chromosome 7A	CGACGACATTAGGAATATGGGAT	TGCGTGTGGGTGTGCTTA	402	58

### Whole genome amplification of single chromosomes

DNA amplification of single chromosomes was performed by MDA using a GE Healthcare GenomiPhi V2 kit (GE Healthcare Life Sciences, Little Chalfont, UK) according to Cápal et al. [15]. Five individual chromosomes were flow-sorted into five 0.2 ml PCR tubes from green sort gates from each HOSUT line and their DNA amplified. The amplified DNA was evaluated on 1.5 % agarose gel, purified using magnetic beads (AMPure XP system, Beckman Coulter, Inc., Brea, CA, USA) and the concentration was measured by a spectrophotometer (NanoDrop, Thermo Fisher Scientific Inc., Waltham, MA, USA).

## Additional files

10.1186/s13007-016-0124-8 Flow karyotypes (histograms of fluorescence intensity) obtained after the analysis of DAPI-stained chromosomes isolated from three transgenic lines and cv. Chinese Spring of common wheat. Flow karyotypes of the transgenic lines are indistinguishable from each other, and slightly differ in profiles of the major composite peaks from those of Chinese Spring.
10.1186/s13007-016-0124-8 Verification of marker specificity. PCR with a full set of chromosome-specific wheat STS markers was performed using genomic DNA of cv. Chinese Spring and corresponding nullitetrasomic lines for chromosomes 1A, 4A, 5D and 7A. The markers, which resulted in amplification products only in Chinese Spring and not in the nullitetrasomic lines, were used in this study.
