# Supplementary figures and images for: The utility of flow sorting to identify chromosomes carrying a single copy transgene in wheat
Source: Plant Methods. 2016 Apr 25;12:24. doi: 10.1186/s13007-016-0124-8 (PMC4845436; doi:10.1186/s13007-016-0124-8)

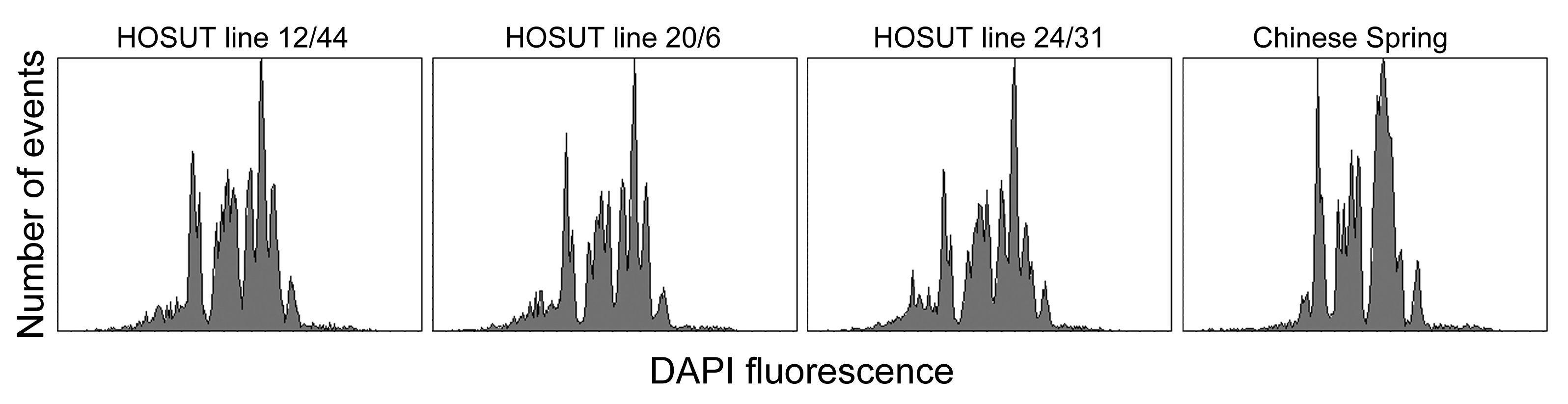

Supplement: Supplementary file 1 — 10.1186/s13007-016-0124-8 Flow karyotypes (histograms of fluorescence intensity) obtained after the analysis of DAPI-stained chromosomes isolated from three transgenic lines and cv. Chinese Spring of common wheat. Flow karyotypes of the transgenic lines are indistinguishable from each other, and slightly differ in profiles of the major composite peaks from those of Chinese Spring. [file 13007_2016_124_MOESM1_ESM.tif]

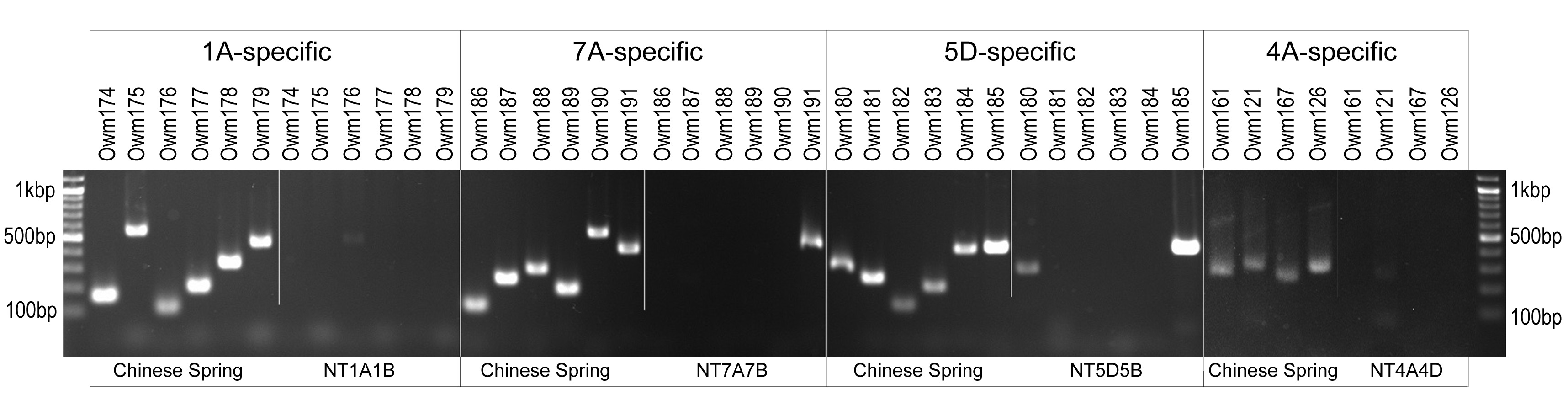

Supplement: Supplementary file 2 — 10.1186/s13007-016-0124-8 Verification of marker specificity. PCR with a full set of chromosome-specific wheat STS markers was performed using genomic DNA of cv. Chinese Spring and corresponding nullitetrasomic lines for chromosomes 1A, 4A, 5D and 7A. The markers, which resulted in amplification products only in Chinese Spring and not in the nullitetrasomic lines, were used in this study. [file 13007_2016_124_MOESM2_ESM.tif]
